# Fulminanter Verlauf einer Ellenbogenschwellung

**DOI:** 10.1007/s00104-025-02403-y

**Published:** 2025-10-30

**Authors:** Paulina Marie Dorten, Michael J. Raschke, Tobias Hirsch, David Kampshoff, Dagmar Horn, Josef Stolberg-Stolberg

**Affiliations:** 1 Klinik für Unfall‑, Hand- und Wiederherstellungschirurgie, Universitätsklinikum Münster, Albert-Schweitzer-Campus 1, Gebäude W1, 48149 Münster, Deutschland; 2 Geschäftsbereich Apotheke, Universitätsklinikum Münster, Münster, Deutschland; 3 Klinik für Plastische Chirurgie, Universitätsklinikum Münster, Münster, Deutschland

## Teil 1

### Anamnese und klinischer Befund

Eine 47-jährige Patientin stellte sich extern mit progredienter, schmerzhafter Schwellung am linken Ellenbogen vor, lokalisiert im Bereich der Bursa olecrani. Ein vorausgehendes Trauma oder erinnerliches Ereignis wurde verneint. Es bestanden zunehmende Schmerzen, Rötung, Überwärmung und eine deutliche Bewegungseinschränkung des Gelenks. Eine ähnliche Episode ließ sich anamnestisch nicht eruieren. Nebenbefundlich bestand lediglich eine Hypothyreose. Im Verlauf desselben Tages kam es zu rascher Größenzunahme der Schwellung und klinischer Verschlechterung mit hämodynamischer Instabilität. Eine initiale CT-Untersuchung ergab weder Gasbildung noch Abszedierung. Bei Übernahme in unsere Klinik zeigte die Patientin folgende Laborwerte: CRP 179 mg/l, Leukozyten 12,86 x 10^9/l, Procalcitonin 9,88 µg/l; ohne Fieber.

### Weiteres Procedere

Nach initialer ambulanter Behandlung mit Ampicillin/Sulbactam, das wegen Allergie abgesetzt werden musste, kam es rasch innerhalb von Stunden zu massiver Schwellung von Ober- und Unterarm. Bei beginnender Sepsis erfolgte die stationäre Aufnahme und Umstellung der Antibiotikatherapie auf Meropenem und Clindamycin [4]. Aufgrund des initial klinischen Verdachts einer nekrotisierenden Fasziitis wurden mehrere Operationen durchgeführt: langstreckige Fasziektomie, Bursektomie sowie temporäre Weichteildeckung [1, 2, 4]. Blutkulturen blieben steril.

**Abb. 1:**
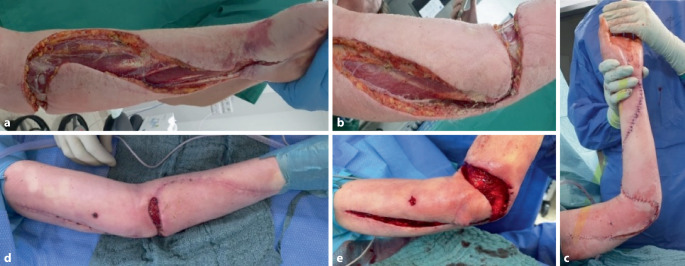
Klinischer Verlauf: intraoperativer Befund nach initialer Bursektomie und langstreckiger Fasziektomie (**a**, **b**). Partieller sekundärer Wundverschluss (**c**). Rezidiv mit Fistelporus über dem Epicondylus radialis und ausgedehnten Kolliquationsnekrosen der Subkutis (**d**, **e**)

Im Verlauf entwickelte die Patientin eine manifeste Sepsis mit hämodynamischer Instabilität, sodass eine intensivmedizinische Betreuung erforderlich wurde. Wiederholte Débridements mit Anlage eines Vakuumverbandes (Abb. 1a–c) folgten. Aus allen intraoperativ gewonnenen Gewebeproben ließ sich *Streptococcus pyogenes*, in einer zusätzlich *Staphylococcus haemolyticus*, nachweisen. Nach Rücksprache mit unserem Antibiotic-Stewardship(ABS)-Team wurde zunächst die bestehende Kombination beibehalten, anschließend Clindamycin als Monotherapie fortgeführt. Unter kontinuierlicher Wundkonditionierung stabilisierten sich die Verhältnisse, sodass nach 2 Wochen ein plastisch-chirurgischer Wundverschluss erfolgen und die antibiotische Therapie beendet werden konnte. Die Patientin wurde anschließend entlassen.

Drei Wochen später stellte sie sich mit rezidivierender Infektion erneut vor. Klinisch zeigte sich ein Fistelporus über dem Epicondylus radialis bei liegendem Fadenmaterial. Intraoperativ wurden Fistelexzision, radikales Débridement und Exploration des Ellenbogengelenks durchgeführt (Abb. 1d, e). Es erfolgte eine ausgedehnte Probenentnahme, anschließend Anlage von Vakuumverband und Oberarmschiene. Erneut konnte *Streptococcus pyogenes* nachgewiesen werden, woraufhin nach erneuter Rücksprache mit unserem ABS-Team eine gezielte, resistenzgerechte Therapie mit oralem Linezolid eingeleitet wurde. Zur Ruhigstellung und weiteren Weichteilkonditionierung wurde ein ellenbogengelenkübergreifender Fixateur externe angelegt.

## Wie lautet Ihre Diagnose?

## Teil 2

### Definition

Die septische Bursitis ist eine infektiöse Entzündung des Schleimbeutels, meist durch *Staphylococcus aureus* (80–90 % der akuten Fälle) oder Streptokokken verursacht [5]. Erreger gelangen typischerweise über Mikrotraumen, Hautläsionen oder durch direkte Ausbreitung aus benachbarten Infektionen (per continuitatem) in die Bursa; bei tief gelegenen Bursae ist auch eine hämatogene Streuung möglich [1]. Besonders betroffen sind oberflächliche mechanisch exponierte Bursae wie die Bursa praepatellaris oder Bursa olecrani. Klinisch zeigen sich Schwellung, Druckschmerz, Rötung und Überwärmung, systemische Symptome wie Fieber fehlen jedoch häufig. Da sich die Symptomatik nicht immer sicher von einer aseptischen Bursitis abgrenzen lässt, ist zur Diagnosesicherung die Aspiration der Bursaflüssigkeit mit Zellzahl, Gram-Präparat und Kultur essenziell.

### Abschließend therapeutisches Vorgehen

**Abb. 2:**
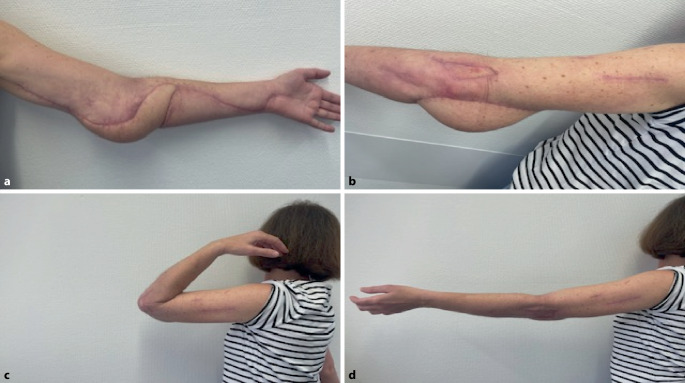
Ambulante Verlaufskontrolle 3 Monate nach Lappendeckung: mediale Ansicht (**a**) und laterale Ansicht (**b**). Funktionelles Ergebnis Beugung (**c**) und Streckung (**d**)

Im weiteren Verlauf führte der erneute Gewebeuntergang zu freiliegendem Epicondylus ulnaris, sodass eine lappenplastische Deckung notwendig wurde. Der Bandapparat war allzeit intakt. Nach Konditionierung erfolgte die Defektdeckung mittels freiem „anterolateral thigh flap“ (ALT). Der Gefäßanschluss wurde End-zu-Seit an die A. brachialis, venös an Begleitvene und V. basilica vorgenommen. Durch die Wahl der Lappenplastik konnte ein vollwertiger Gewebeersatz mit guter Abdeckung der knöchernen Strukturen und vollumfänglicher Beweglichkeit im Gelenkbereich sowie originärer und stabiler Haut und ein schlankes Lining erzielt werden. Die Wahl einer fasziokutanen Lappenplastik verhindert den Verlust einer funktionellen Einheit im Bereich der Entnahmestelle wie im Fall einer transplantierten Muskellappenplastik und geht mit einer geringen Hebemorbidität einher [3]. Intraoperativ entnommene Proben blieben steril. Nach Entfernung des Fixateur externe zeigte sich die Lappenplastik vital und die Patientin konnte in die ambulante Weiterbehandlung entlassen werden (Abb. 2a–d). Linezolid wurde für 6 Wochen unter regelmäßiger Kontrolle der Serumspiegel und Organfunktionen fortgeführt.

**Diagnose:** Septische Bursitis olecrani mit Entwicklung einer nekrotisierenden Weichteilinfektion

## Fazit für die Praxis

Zusammenfassend lassen sich aus diesem Fall folgende Aspekte ableiten:

- Rasche Progredienz sollte frühzeitig Anlass zur erweiterten Diagnostik und stationären leitliniengerechten Therapie geben. Zunehmende Schwellung, Schmerzen, Überwärmung und Bewegungseinschränkung erfordern erhöhte Wachsamkeit gegenüber fulminanten Verläufen.
- In komplexen Infektionsverläufen ist die enge interdisziplinäre Zusammenarbeit essenziell – insbesondere Unfallchirurgie, Anästhesiologie, Intensivmedizin, Mikrobiologie, Infektiologie und plastische Chirurgie. Die zeitgerechte Einbindung dieser Disziplinen ermöglicht eine abgestimmte operative, medikamentöse und supportive Versorgung.
- Entzündungsparameter, Wundverhältnisse und klinische Stabilität müssen engmaschig abhängig vom klinischen Verlauf überwacht und dokumentiert werden. Bei Reinfektion ist eine frühzeitige Therapieanpassung mit ggf. operativer Revision entscheidend für den Behandlungserfolg.
